# Case Report: Neuroendocrine Tumor With Cardiac Metastasis

**DOI:** 10.3389/fcvm.2020.596921

**Published:** 2020-12-04

**Authors:** Rachel E. Kinney, Robert Decker, Deborah Sundlof, Muhammad A. Rizvi, Kelly Schadler

**Affiliations:** Lehigh Valley Health Network, Allentown, PA, United States

**Keywords:** neuroendocrine tumor, intracardiac metastases, ileal neuroendocrine tumor, infiltrative myocardial metastasis, ^177^Lu DOTATATE

## Abstract

Neuroendocrine tumors (NETs), also known as carcinoid tumors, are a heterogeneous group of neoplasms that arise from cells throughout the neuroendocrine system, most commonly arising from the gastrointestinal (GI) tract, lungs, and bronchi. Myocardial carcinoid metastasis is rare with an incidence among metastatic carcinoid patients of 4%. They are generally asymptomatic and detected incidentally. Infiltrative myocardial metastasis secondary to carcinoid tumor is exceedingly rare with only single-digit cases reported in the literature. We report the case of a 65-years-old female with a newly diagnosed ileal neuroendocrine tumor as well as heart failure due to infiltrative myocardial metastasis.

## Introduction

Neuroendocrine tumors (NETs) are a heterogeneous group of neoplasms that arise from cells throughout the neuroendocrine system, most commonly arising from the gastrointestinal (GI) tract, lungs, and bronchi. They are classified into several broad groups including carcinoid tumors (encompassing NETs of the GI tract, lung, and thymus), NETs of the pancreas, NETs of unknown primary, adrenal gland tumors, pheochromocytoma/paraganglioma, and extrapulmonary poorly differentiated neuroendocrine carcinoma. Further, these can be subclassified based on location and functional status (1). The overall annual incidence of NETs is estimated to be 2.5–5 per 100,000 with two thirds of those representing carcinoids and the other third being other NETs (2). Of the carcinoid tumors, the most common location is within the small bowel, and in particular within the ileum (3). When metastatic disease is present, it is most commonly within the liver. Myocardial carcinoid metastasis is rare with an incidence among metastatic carcinoid patients of 4% and in general are asymptomatic and detected incidentally (4). Of myocardial metastatic disease, 40% involve the right ventricle, 53% involve the left ventricle, and 7% involve the septum. The majority of these lesions are seen as non-infiltrative and appear as homogenous masses (5). Review of the literature reveals only one previously reported case of infiltrative myocardial metastasis secondary to carcinoid tumor (6). This case describes the exceedingly rare presentation of a patient found to have symptomatic infiltrative cardiac metastases from a primary ileal carcinoid tumor.

## Case Report

**Figure 1:**
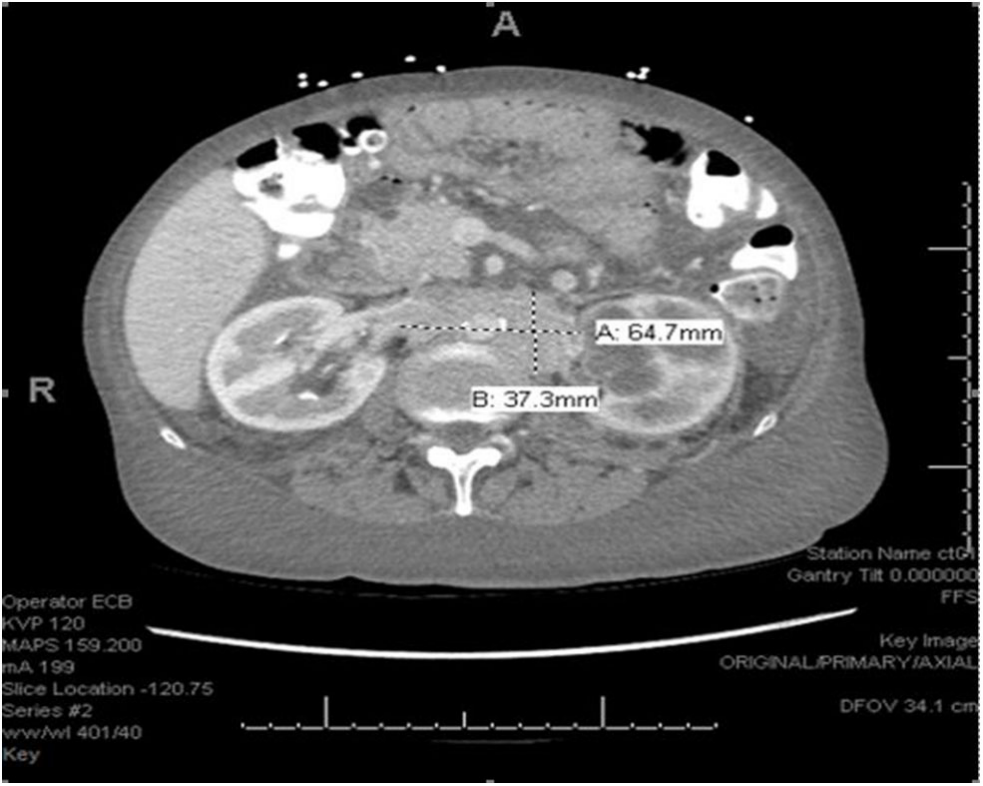
CT scan with extensive retroperitoneal adenopathy.

We describe the case of a 65-years-old female with no significant past medical history who was admitted to the hospital with complaints of lower extremity swelling and dyspnea on exertion. She also admitted to a 50 pound unintentional weight loss over the 3 years prior to admission. A CT of the chest, abdomen and pelvis with contrast (Figure 1) revealed extensive retroperitoneal adenopathy with the largest conglomerate of lymph nodes at the level of the renal hila measuring 6.5 × 3.7 cm with resultant severe left hydroureteronephrosis. Diffuse anasarca was also noted with small bilateral pleural effusions and a small pericardial effusion. Evaluation of the gastrointestinal tract revealed distorted mural thickening of the distal and terminal ileum as well as the proximal ascending colon.

The patient underwent a left retroperitoneal lymph node core biopsy which revealed a well-differentiated neuroendocrine tumor from a gastrointestinal tract primary site. Immunoperoxidase stains revealed positive CAM 5.2, chromogranin, synaptophysin, CD56, CDX2 and ER with a Ki 67 < 2%. In contrast, CK 7, CK 20, TTF-1, P40, p63, CA 125, WT1, BRST2, CA 19.9, PAX8, GATA 3 were all negative. There was no immunophenotypic evidence of a clonal B or T-cell population. Chromogranin and synaptophysin are the two most common markers for neuroendocrine cells and CDX2 is highly sensitive and specific for a GI tract origin. Further, the low Ki 67 supports the diagnosis of a low-grade, well-differentiated tumor, such as a carcinoid or neuroendocrine tumor.

Labwork was significant for chromogranin A level of 6,620 ng/ml (reference range 0–95 ng/mL) and a urine 5-HIAA level of 49 mg/gCR (reference range 0–14 mg/gCR). Gastrin level was within normal limits at 35 pg/mL (reference range 0–100 pg/mL).

**Figure 2:**
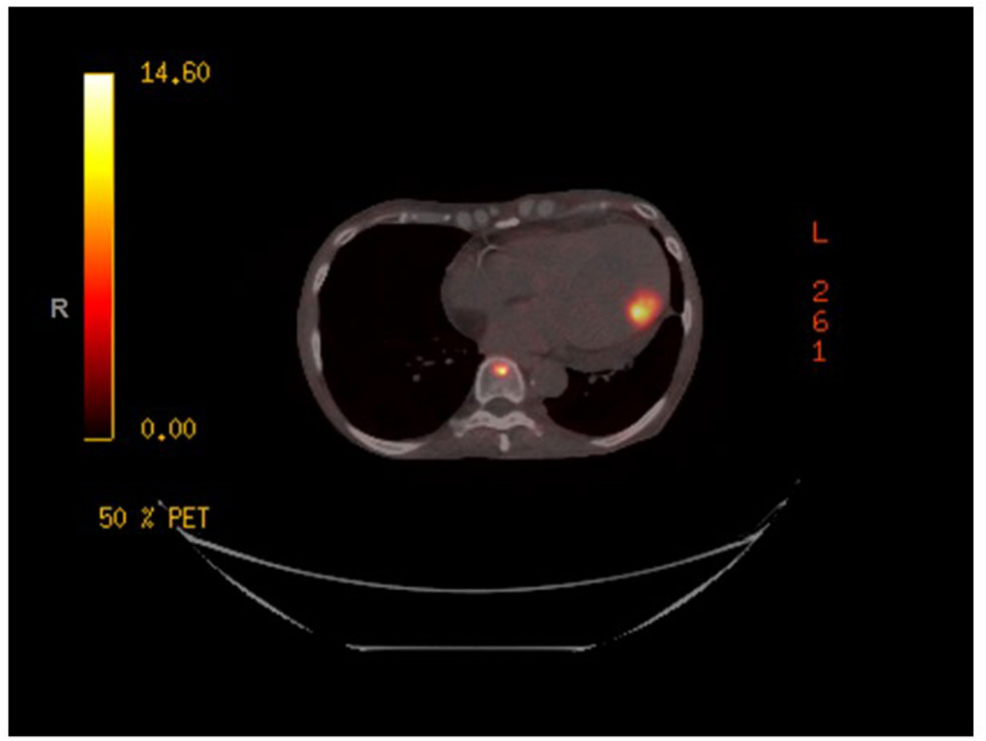
Gallium-68 dotatate PET/CT scan demonstrating avidity in the left ventricular myocardium.

A gallium-68 dotatate PET/CT scan (Figure 2) was performed utilizing the Krenning Scoring System for visual grading of pathologic Gallium-68 Dotatate radiotracer uptake as noted below:

Score = 0 (no uptake)
Score = 1 (very low uptake)
Score = 2 (less than or equal to liver uptake)
Score = 3 (greater than liver uptake)
Score = 4 (greater than spleen uptake)

The study revealed a primary somatostatin-avid tumor within the distal ileum with a Krenning score of 3. There was evidence of somatostatin avid metastatic disease in the left ventricular myocardium, liver, and in multiple lymph node groups below the diaphragm with activity greater than liver, Krenning score of 3. Additionally, there was evidence of somatostatin avid osseous metastases within the skull, axial and proximal appendicular skeleton with the majority of the lesions demonstrating activity greater than liver, Krenning score of 3. However, there was one lesion in the bone marrow of the proximal shaft of left femur demonstrating activity greater than spleen, Krenning score of 4.

**Figure 3:**
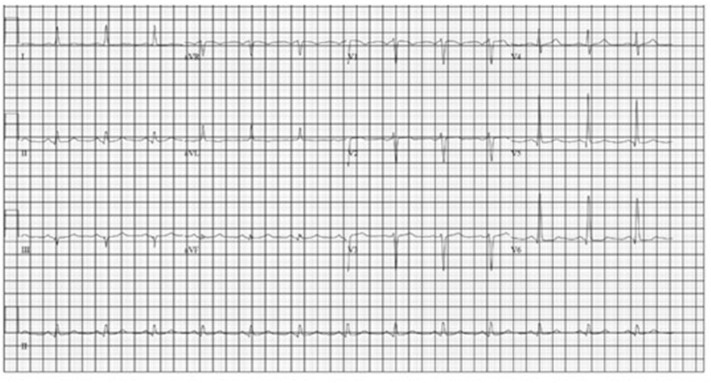
First EKG (11/12/18).

A transthoracic echocardiogram revealed moderate concentric left ventricular hypertrophy with mild global hypokinesis, severe diastolic dysfunction with a mitral E/E' ratio of 19.8 (indicative of significant diastolic dysfunction, normal ratio <8) and mild LV systolic dysfunction with an ejection fraction of 45–50%. The myocardium had a speckled appearance which raised the concern for infiltrative cardiomyopathy. Additional labwork was obtained and included an NT-proBNP, which was markedly elevated at 11,660 pg/ml (reference range <125 pg/mL) and a troponin I level, which was normal at <0.02 ng/ml. In response, serum protein electrophoresis with immunofixation was performed and revealed no evidence of a monoclonal protein. Additionally, kappa and lambda free light chain quantification revealed a normal ratio. A technetium-^99^m pyrophosphate cardiac scan revealed no evidence of transthyretin-related cardiac amyloid with only very mild cardiac radiotracer uptake and a calculated mean heart to contralateral thoracic ratio of ~1.3. An electrocardiogram (ECG) initially revealed normal sinus rhythm, as pictured in Figure 3.

**Figure 4:**
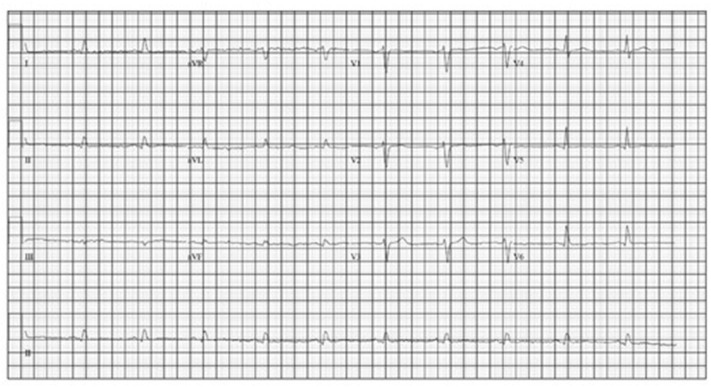
Second EKG (4/14/19).

She was started on monthly octreotide 20 mg injections with improvement in her Chromagranin A levels from 6,620 to 2,640 after 2 cycles. However, her chromogranin A level after cycle 4 had increased slightly to 2,969. Shortly after her 5th treatment she was again admitted with an acute diastolic heart failure exacerbation after she was noted to be hypoxic and dyspneic in her home. An echocardiogram showed improvement in her systolic function with an ejection fraction of 60–70%, however severe concentric left ventricular hypertrophy (LVH) with a speckled appearance was noted, consistent with her known infiltrative cardiomyopathy secondary to metastatic neuroendocrine tumor. A repeat EKG (Figure 4) revealed lower voltage complexes in comparison to her initial EKG as well as worsening prolongation of the QRS, indicating progressive infiltrative disease. While inpatient, she was treated with intravenous diuretics and underwent thoracentesis with improvement in her symptoms.

After discharge she was briefly maintained on long-acting octreotide injections but due to a significant functional decline with progressive cachexia and ongoing heart failure symptoms she opted for hospice care.

## Discussion

Intracardiac NET metastasis is rare and has been estimated to occur in <5% of patients with neuroendocrine tumors. Additionally, they are usually asymptomatic and are detected incidentally with enhanced imaging modalities (5). The use of gallium-68 dotatate PET/CT imaging has increased the ability to detect cardiac metastases in both ileal and non-ileal NETs (7, 8). Calissendorf et al. presented a case series of 4 patients at their institution with myocardial metastases out of a total of 92 patients with NETs. They reported the incidence of NET with cardiac metastases to be 4.3%, which was slightly higher than the previously reported incidence of 2–4% (7). A larger case series with a review of the literature was published in 2016 by Bonsen et al. and described six patients with cardiac NET metastases detected by gallium-68 dotatate PET/CT. The overall incidence of cardiac metastasis from NET was 2.2% and as noted in other series, cardiac symptoms were not observed in this cohort. Table 1 provides an overview of published case reports on cardiac NET metastases.

**Table 1:** Review of case reports on cardiac NET metastases.

**Authors**	**Year**	**Incidence**	**Cardiac symptoms**
Yeung et al.	1996	1	Yes
Patel et al.	2009	1	No
Amrani-Raissouni et al.	2011	1	No
Khangembaum et al.	2012	1	No
Williamson et al.	2013	1	No
Carerras et al.	2013	29 out of 4,210	N/A
Calissendorff et al.	2013	4 out of 92	N/A
Bonsen et al.	2016	6	No

While nearly all patients with cardiac metastases from NETs remain asymptomatic from a cardiac perspective, our patient presented with heart failure at the time of her diagnosis. Additionally, with progression of her disease she also had progressive EKG changes felt to be due to intra-cardiac metastases resulting in an infiltrative cardiomyopathy. An EKG comparison from the time of diagnosis to the time of her 2nd hospital admission for acute heart failure exacerbation revealed further prolongation of the QRS complex as well as progressively lower voltage, indicating progressive myocardial infiltration (Figures 3, 4).

It is important to note that while our patient had cardiac metastases, she had no evidence of carcinoid heart disease. This is in contrast to the Mayo Clinic case series that identified 11 patients with intracardiac metastases, eight of which had carcinoid heart as well (5).

The treatment of cardiac NET metastases is not standardized due to the rarity of the condition and given the often asymptomatic presentation. Multiple therapy options exist for NETs including octreotide or biologically targeted therapies, such as sunitinib or everolimus. Cytotoxic chemotherapy is typically not utilized due to limited response rates. Additionally, cardiotoxic regimens including doxorubicin or daunorubicin may further compromise cardiac function. Radiolabeled octreotide therapy, such as peptide receptor radionuclide therapy (PRRT) is an emerging treatment for NETs and may play a role in the future for patients with cardiac metastases as part of their systemic therapy (1). Makis et al. reviewed 251 patients with neuroendocrine tumors treated with ^177^Lu DOTATATE peptide receptor radionuclide therapy or ^131^I-MIBG. Of those 251 patients, two had cardiac metastases. The first of these patients was treated with three cycles of ^131^I-MIBG of 200 mCi, each given 8–16 weeks apart. This patient went on to achieve stable disease for 9 months before being diagnosed with progressive liver metastasis on CT. He died 3 years later due to a secondary unrelated malignancy, end-stage poorly differentiated esophageal adenocarcinoma. The second patient in this study with cardiac metastasis received ^177^Lu DOTATATE peptide receptor radionuclide therapy (Lutathera). The patient received four cycles of ^177^Lu DOTATATE of 150 mCi each 8–12 weeks apart and went on to achieve stable disease for 20 months before passing away from failure to thrive (9).

## Conclusion

While intracardiac metastases from NETs is exceeding rare, when they are present it is typically picked up incidentally and causes no clinic symptoms. After an extensive literature search, our case illustrates the rare case of symptomatic myocardial metastases with diffuse infiltration and resultant symptomatic cardiomyopathy.

## Data Availability Statement

The original contributions presented in the study are included in the article/supplementary material, further inquiries can be directed to the corresponding author/s.

## Ethics Statement

Written informed consent was not obtained from the individual(s) for the publication of any potentially identifiable images or data included in this article.

## Author Contributions

RK, RD, MR, DS, and KS were involved in the writing and editing of the manuscript. All authors contributed to the article and approved the submitted version.

## Conflict of Interest

The authors declare that the research was conducted in the absence of any commercial or financial relationships that could be construed as a potential conflict of interest.
